# Developmental dyscalculia: compensatory mechanisms in left intraparietal regions in response to nonsymbolic magnitudes

**DOI:** 10.1186/1744-9081-5-35

**Published:** 2009-08-05

**Authors:** Liane Kaufmann, Stephan E Vogel, Marc Starke, Christian Kremser, Michael Schocke, Guilherme Wood

**Affiliations:** 1 Department of Psychology, University of Salzburg, Salzburg, Austria; 2 Department of Pediatrics IV, Section Neuropediatrics, Medical University Innsbruck, Innsbruck, Austria; 3 Department of Psychology, University of Innsbruck, Innsbruck, Austria; 4 Department of Radiology I, Medical University Innsbruck, Innsbruck, Austria; 5 Center for Neurocognitive Research, University of Salzburg, Salzburg, Austria

## Abstract

**Background:**

Functional magnetic resonance imaging (fMRI) studies investigating the neural mechanisms underlying developmental dyscalculia are scarce and results are thus far inconclusive. Main aim of the present study is to investigate the neural correlates of nonsymbolic number magnitude processing in children with and without dyscalculia.

**Methods:**

18 children (9 with dyscalculia) were asked to solve a non-symbolic number magnitude comparison task (finger patterns) during brain scanning. For the spatial control task identical stimuli were employed, instructions varying only (judgment of palm rotation). This design enabled us to present identical stimuli with identical visual processing requirements in the experimental and the control task. Moreover, because numerical and spatial processing relies on parietal brain regions, task-specific contrasts are expected to reveal true number-specific activations.

**Results:**

Behavioral results during scanning reveal that despite comparable (almost at ceiling) performance levels, task-specific activations were stronger in dyscalculic children in inferior parietal cortices bilaterally (intraparietal sulcus, supramarginal gyrus, extending to left angular gyrus). Interestingly, fMRI signal strengths reflected a group × task interaction: relative to baseline, controls produced significant *de*activations in (intra)parietal regions bilaterally in response to number but not spatial processing, while the opposite pattern emerged in dyscalculics. Moreover, beta weights in response to number processing differed significantly between groups in left – but not right – (intra)parietal regions (becoming even positive in dyscalculic children).

**Conclusion:**

Overall, findings are suggestive of (a) less consistent neural activity in right (intra)parietal regions upon processing nonsymbolic number magnitudes; and (b) compensatory neural activity in left (intra)parietal regions in developmental dyscalculia.

## Background

Calculation difficulties lead to considerable disadvantages in academic and occupational activities. Developmental dyscalculia describes severe calculation difficulties despite average intellectual abilities and good schooling [1]. Developmental dyscalculia is not a unitary dysfunction but may reflect quite heterogeneous performance profiles [2]. Nonetheless, impaired number magnitude (i.e., numerosity) processing should be considered a key cognitive deficit of developmental dyscalculia [3-5].

Parietal structures are known to subserve specific aspects of number processing [6]: First, number magnitude processing per se is mediated by the intraparietal sulcus (IPS) bilaterally, as confirmed by functional imaging (dyscalculic adults with Turner syndrome [7], structural imaging (dyscalculic adolescents born prematurely [8] and transcranial magnetic stimulation (healthy adults [9]). Second, the bilateral posterior superior parietal lobe (PSPL) subserves spatial attention on the mental number line (according to which mental number representations are spatially oriented from left to right). Third, the left angular gyrus (AG) supports verbally mediated number processing such as overlearnt number fact retrieval (2+4 = 6, 2 × 4 = 8).

The still scarce developmental functional Magnetic Resonance Imaging (fMRI) studies suggest that even preschool children without prior formal math education recruit parietal regions upon solving numerical tasks [10]. However, the neural correlates of number magnitude processing differ between mature and developing brain systems. For instance, Rivera and colleagues [11] report a positive correlation between age and (intra)parietal activations, which has been interpreted as reflecting increasing functional specialization of parietal cortex for number processing [see also [11,13]]. Upon comparing children with and without dyscalculia, Kucian et al. [14] report a positive correlation between performance accuracy on tasks thought to tap approximate calculation (e.g., 3+8 = 10 or 5?) and fronto-parietal activations (being most accentuated in the left IPS, the right middle and left inferior frontal gyrus). Recent findings of Mussolin et al. [15] and Kaufmann et al. [16] further support the notion that dyscalculic children produce aberrant IPS activation upon solving *symbolic *number tasks (i.e., comparison and ordinality judgments on Arabic numbers, respectively). As regards the involvement of the IPS in *nonsymbolic *number magnitude classifications, the literature is controversial: deficient recruitment of the right IPS in developmental dyscalculia is reported by Price and colleagues [17], while Kucian et al. [14] report comparable parietal activations in children with and without dyscalculia. Nonetheless, the absence of group differences in the nonsymbolic number comparison task is rather surprising upon considering the above-mentioned strong evidence being suggestive of a key role of the IPS for number magnitude processing.

Main aim of the present study is to compare functional activation patterns in response to nonsymbolic number processing in children with and without dyscalculia.

## Methods

### Participants

From 22 children initially tested 4 were excluded due to excessive motion in the scanner (see section *fMRI acquisition *below). Thus, 18 children (9 with dyscalculia, 9 controls) participated in the study. Groups were matched regarding age (*t*(16) = -0.171; *n.s*.; mean age children with dyscalculia 9.6 years/SD 1.1 vs. controls 9.7 years/SD 1.6) and intellectual level (*t*(16) = -1.068; *n.s*.; abbreviated German version of the WISC-III [18]: dyscalculic children 99.6/SD 11.4 vs. controls 108.5/SD 22.2). Participating children attended grades 2 to 4. Thus, it can be assumed that they easily master the experimental task requiring them to make a simple numerical classification on finger patterns (see below). Children with a history of neurological and/or psychiatric disorders were excluded. Further exclusion criteria were the presence of attention deficit disorder, dyslexia, sensory and motor impairments (possibly interfering with test compliance) and medication intake. Developmental dyscalculia was defined as a significant (1.5 SD) discrepancy between average IQ (>85) and below average performance (<40 t-score) on a standardized dyscalculia test (German dyscalculia test Heidelberger Rechentest/HRT [19]). Importantly, group differences were significant as regards magnitude processing skills (corresponding HRT subtest; *t*(16) = -9.035; *p *< .001; mean T-score children with dyscalculia 34.8/SD 4.2 vs. controls 65.3/SD 9.2). The magnitude processing subtest of the HRT requires children to judge which of two Arabic numbers is the numerically larger one or alternatively, whether the two numbers are equal. Later on in the test, the level of difficulty increases and one or both Arabic numbers are replaced by simple equations (e.g., 9-2 > 6). The research project was approved by the local ethical committee. All participants and their caregivers had given written informed consent. Children received a small monetary compensation for study participation.

### Experimental task

The experimental design was identical to the one employed in a previously published developmental fMRI study [13] and required children to make numerical, spatial and color judgments. Here we will focus on number and spatial processing as both domains are supported by (intra)parietal regions [20] and thus, subtracting activations obtained upon spatial processing from those obtained upon number processing ensures that only number-specific activations remain. Upon viewing two horizontally presented hands children were asked to indicate by button press (i) on which side more fingers are raised (nonsymbolic numerical task); and (ii) whether the orientation of the palms (spatial control task) are identical (right button) or not identical (left button). Importantly, identical stimuli were used across the experimental task conditions, varying task instructions only. Numerical differences (distances 1 to 4) and spatial orientation (identical vs. non-identical) were matched. Side of presentation was counterbalanced across tasks. Behavioral practice outside the scanner ensured task comprehension. Tasks were presented in box-car fashion (4 blocks per task, 6 stimuli per block; stimulus duration 4000 msec; inter-stimulus-interval 1000 msec). Experimental blocks were interspersed with rest blocks (12000 msec each) and preceded by an instruction picture that was defined as predictor of no interest (3000 msec). Stimuli subtended a visual angle of 5.7° (width) and 2.5° (height). Within blocks, stimuli were presented in randomized order. Task order was counterbalanced across participants. Same-task conditions were presented consecutively to minimize cognitive switching demands between task instructions, what is especially important in children. Within blocks, stimuli were presented in randomized order. Task order was counter-balanced across participants. Psychometric testing (intelligence, arithmetic) and brain imaging were undertaken on two separate days.

### fMRI acquisition

Images were acquired with a 1.5 Tesla whole-body system (Siemens Magnetom, Avanto). For the functional measurements a T2-weighted echo-planar/EPI sequence was used (TR = 3060 ms, TE = 50 ms, flip angle = 90°, FOV = 220 mm, 64 × 64 matrix, 32 axial slices, voxel size = 3.4 × 3.4 × 3 mm). 209 functional images were acquired. In each scanning session a high-resolution T1-weighted 3-dimensional volume (voxel size = 1 × 1 × 1 mm) for coregistration and anatomical localization was acquired.

Data analysis was performed using Brain Voyager QX1.9.

### Preprocessing

Functional images of each subject were motion corrected (linear trend removal; high-pass filter). Coregistration and Talairach transformation (trilinear interpolation [21]) of the functional images were performed using the acquired T1-weighted three-dimensional (3D) volumes. The 3D-aligned time course data were smoothed with a 4 mm FWHM kernel.

### Statistics

Individuals with excessive head motion were excluded from analysis (cut-off criteria: deviations exceeding 3 mm; sudden jumps greater than 2 mm). Activation coefficients for the experimental conditions were estimated for each individual in within-subjects models (including motion parameters as regressors) and compared statistically with *t*-contrasts in between-subjects GLM designs. All images depict activations obtained at *p *< 0.001, uncorrected (*p *< 0.05, corrected at cluster level).

## Results

### Behavior

As regards behavioral data collected during scanning, overall accuracy rates were high (both groups > 90% correct) and comparable between groups (*t*(16) = .436; *n.s*.). Likewise, group differences did not become significant as regards overall response latencies (*t*(16) = -1.366; *n.s*.). Both groups achieved the lowest error rates and quickest responses in the numerical task (Table 1). Although main effects of tasks were significant with respect to accuracy (*F*(1,16) = 7.681; *p *< .05) and reaction time (*F*(1,16) = 68.544; *p *< .001), the interactions group × task did not become significant (accuracy *F*(1,16) = .957; *n.s*.; reaction time *F*(1,16) = .522; *n.s.*) thus revealing comparable performance patterns between groups.

**Table 1:** Mean response accuracies and reaction times for children with developmental dyscalculia and controls on the two tasks.

	**Children with dyscalculia**		**Children without dyscalculia**	
	
	Response accuracy in %	Reaction times in msec	Response accuracy in %	Reaction times in msec
Nonsymbolic numerical judgments	95.4 (6.4)	1170.7 (237.7)	94.0 (11.0)	1002.3 (257.4)
Spatial judgments	84.7 (8.8)	1722.1 (426.4)	88.9 (9.3)	1465.3 (446.5)

Standard deviations are given in parenthesis.

### Imaging

*Baseline contrasts *(experimental vs. rest conditions) revealed largely comparable activation patterns between the two groups (uncorrected *p *< .001). Dyscalculic and to a lesser extent control children exhibited considerable interindividual differences in activation patterns. Beyond occipital activations (reflecting visual processing), activations in fronto-parietal regions were observed in both groups in all task conditions. Only few activation differences between groups were found in the average activation over all tasks: dyscalculic children produced significantly stronger activations than controls (i) in and around the left IPS (TC: -39, -49, 46; mean TC for IPS as reported by Cohen Kadosh and collaborators [22]: -31, -50, 45/37, -46, 42; standard deviations 8, 9, 7/9, 11, 6) and right inferior and superior frontal gyrus (TC: 45, 5, 19; TC: 39, 47, 25) in response to number processing; and (ii) in right postcentral gyrus bordering superior parietal lobe/IPS (TC: 27, -40, 52) upon processing spatial information.

#### Task-specific contrasts (dyscalculic children > controls) in fronto-parietal brain regions

**Figure 1:**
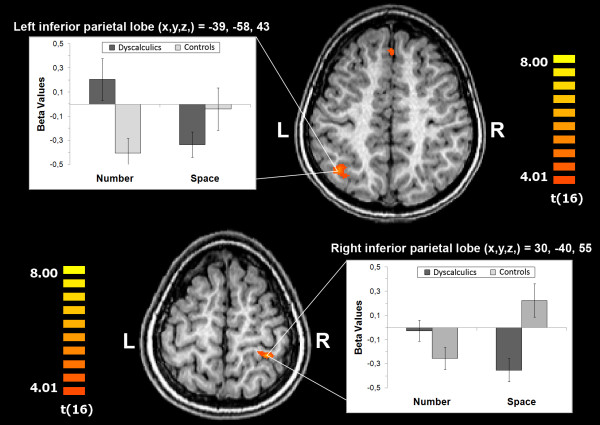
**Talairach coordinates of significant (intra)parietal activation *differences *between children with and without dyscalculia (dyscalculic children > controls) as obtained in the task-specific contrast number processing > spatial processing**. In addition, mean beta-values for both parietal activation foci are plotted separately for task (number and space) and group (dyscalculic children and controls). Error bars denote standard errors of the mean.

Relative to controls, dyscalculic children produced significantly stronger task-specific activations (number > space) in a fronto-parietal network including the IPS and the supramarginal gyrus (SMG) bilaterally (adjoining the left AG; TC: 30, -40, 55; TC: -39, -58, 43; see Figure 1), left postcentral gyrus (TC: -3, -43, 67), paracentral lobe (TC: -6, -25, 49) and right superior frontal gyrus (TC: 1, 32, 44).

Results of post-hoc *t*-tests conducted on the parameter estimates extracted from parietal activation foci (Figure 1) were threefold: First, signal strengths differed significantly between tasks in both groups and in both hemispheres (right IPL [TC: 30, -40, 55]: controls *p *< .001; dyscalculic children *p *< .005; left IPL [TC: -39, -58, 43]: controls *p *< .05; dyscalculic children *p *< .01). Second, task-related activations differed significantly from zero (i.e., baseline), disclosing a group × task interaction: While (de)activations pertaining to numerical processing differed significantly from zero in controls (right IPL *p *< .05; left IPL *p *< .01) but not in children with dyscalculia, (de)activations related to spatial processing differed significantly from zero in dyscalculic children solely (right IPL *p *< .01; left IPL *p *< .01). Finally, beta weights related to number processing differed significantly between groups in the left, but not right IPL (p < .01), while regarding spatial processing group differences reached significance in the right IPL solely (p < .01).

Furthermore, we investigated whether response latencies (upon solving the numerical task in the scanner) correlate with signal strengths in parietal regions of interest as depicted in Figure 1. Correlations calculated for each group separately did not become significant. Across all participants, results disclosed a positive correlation approaching significance between response latencies and signal strength in left IPL only (r = .454; p = .058; right IPL: r = .008; *n.s.*). Likewise, behavioral performance obtained outside the scanner (i.e., magnitude knowledge measured by the corresponding subtest of the standardized dyscalculia test) was negatively correlated to signal strength in left IPL solely (r = -.501; p < .05; right IPL: r = -.324; *n.s.*). Thus, children with slower reaction times and poor magnitude skills (i.e., dyscalculics) produced considerably stronger activations in left IPL.

## Discussion

The present study investigated the neural correlates of nonsymbolic number magnitude processing in 9 year-old children with and without dyscalculia. All dyscalculic children displayed deficient number magnitude (i.e., numerosity) knowledge while control children presented with average numerosity skills as reflected by significant group differences on the respective subtest of the standardized dyscalculia test. Though both groups displayed high and comparable accuracy rates upon performing the rather easy experimental tasks during scanning, dyscalculic children were slower than controls upon making number magnitude classifications, which is in line with the literature [3,4].

Baseline imaging results revealed that both groups recruit comparable fronto-parietal brain regions when making number magnitude classifications. Nonetheless, task-specific activations differed significantly between groups: dyscalculic children producing stronger activations in inferior parietal cortex bilaterally (IPS, SMG, extending to the left AG only). Interestingly, beta weights observed in intraparietal voxels reflected a group × task interaction: While the numerical task led to significant deactivations (relative to baseline) in bilateral IPL in controls but not in children with dyscalculia, the spatial task induced significant bilateral deactivations in dyscalculic children solely. However, upon considering the considerable interindividual variability in signal strengths (i.e., high standard errors, see Figure 1) and the non-significant (relative to baseline) (de)activations in the dyscalculic group in response to number processing, the latter interaction should be interpreted cautiously. Notably, in response to number processing significant group differences emerged in the left IPL only: Children with dyscalculia showed considerably stronger recruitment of left IPL (reflected by a positive fMRI signal), but showed less pronounced deactivations – approaching baseline – in right IPL.

Importantly, our study is the first to report stronger activations in left (intra)parietal regions (including AG) in dyscalculic children relative to controls. Differential activation of the left AG has been previously reported in non-dyscalculic adults upon solving multiplications [23] and in non-dyscalculic children and adults upon solving additions and subtractions [11]. The latter studies report a positive correlation between math competency and signal strength in left AG, possibly reflecting verbally mediated and automatized math fact retrieval [6]. In our study, the significantly elevated left AG activation in dyscalculic children may reflect the presence of (sub)verbal counting strategies. In particular, the presentation of finger patterns may have triggered a counting strategy in dyscalculic children (employed to facilitate processing of the numerical classification task) while control children solved this task without resorting to counting strategies. The latter view is strengthened by our finding of a positive correlation between signal strength in the left IPL and response latency on the numerical comparison task and furthermore, is compatible with previous behavioral studies showing that compared with average calculating peers dyscalculic children display immature counting and calculation strategies [24].

It is important to note that due to different methodological approaches (e.g., different experimental paradigms) a direct comparison across fMRI studies investigating developmental dyscalculia is not feasible. While in the present study children were requested to compare finger patterns, the non-numerical comparison task employed by Kucian et al. [14] and Price et al. [17] required children to classify assemblies of objects and squares (respectively), and yet other studies focused on symbolic number processing involving Arabic digits (number comparison [15]; numerical ordinality [16]). To the best of our knowledge there are no published studies on differences in activation patterns elicited by finger versus dot patterns. Taken together, upon acknowledging that (i) the right IPS preferentially supports nonsymbolic number magnitudes and the left IPS language-related number processing/calculation [25]; that (ii) dyscalculic children have deficient number representations [3,4]; and that (iii) the IPS exerts top-down attentional control [26], it is plausible to assume that the stronger recruitment of left IPL (including AG) observed in our group of dyscalculic children reflects verbally mediated compensatory mechanisms aiming at retrieving deficient/noisy nonsymbolic number representations. Consistent with the latter assumption is our finding of a positive correlation between reaction time and signal strength in left IPL.

Our findings of reduced deactivations in right IPL in developmental dyscalculia are only partially consistent with previous results [15,17]. While in the Price study [17] both groups produced positive beta weights relative to baseline in right IPL (reduced activations in developmental dyscalculia; children taking part in the current study produced negative beta weights relative to baseline in right IPL (reduced deactivations in dyscalculic children). Presently, the cognitive and neural processes underlying deactivations are poorly understood [27]. Given the small sample size and the considerable interindividual variability as regards task-related deactivations in right IPL the present findings need to be interpreted cautiously.

## Limitations of the study

A potential limitation of the study is the small sample size (each group n = 9). Nonetheless, participating children were carefully selected regarding diagnostic criteria, motion artifacts and behavioral performance in the scanner. Thus, we believe that our small, but therefore carefully selected and homogeneous study group may have enhanced the interpretability of our findings.

## Conclusion

Overall, *de*activations observed in right IPL are not fully compatible with previous findings in developmental dyscalculia [15,17] and should be investigated in future studies in more detail. However, the significantly stronger activations in left IPL observed in dyscalculic children are compatible with previous imaging studies investigating aging and cognitive decline [28,29] and may be interpreted as reflecting compensatory mechanisms in left IPL known to support verbally mediated number processing [6]. Our findings are the first to suggest a similar mechanism in developmental dyscalculia: Children with functional deficits need to recruit a wider network of regions to perform the task, and within task-relevant regions, activations are stronger in order to compensate their processing difficulties.

## Abbreviations

AG: angular gyrus; EPI: echo planar imaging; fMRI: functional magnetic resonance imaging; FWHM: full-width half-maximum; IPL: inferior parietal lobe; IPS: intraparietal sulcus; SD: standard deviation; SMG: supramarginal gyrus; TC: Talairach Coordinates.

## Competing interests

The authors declare that they have no competing interests.

## Authors' contributions

LK contributed in developing the study paradigm, recruited subjects, coordinated appointments for fMRI-testing, conducted the behavioral testing, has been involved in fMRI data acquisition and analyses, had a major role in data interpretation and prepared the manuscript. SEV and MS programmed the paradigm and had a major role in fMRI data acquisition and analyses. CK and MS gave substantial technical support in data acquisition and were involved in implementing the fMRI paradigm. GW played a major role in developing the study design, contributed substantially to fMRI data analyses and data interpretation, and contributed to scientific writing by critically reviewing the manuscript. All authors read and approved the final manuscript.

## References

[B1] American Psychiatric Association (1994). Diagnostic and statistical manual of mental disorders.

[B2] Dowker A (2005). Individual differences in arithmetic: Implications for psychology, neuroscience and education.

[B3] Landerl K, Bevan A, Butterworth B (2004). Developmental dyscalculia and basic numerical capacities: A study of 8–9 year-old students. Cognition.

[B4] Rubinsten O, Henik A (2005). Automatic activation of internal magnitudes: a study of developmental dyscalculia. Neuropsychol.

[B5] Wilson A, Dehaene S, Coch D, Dawson G, Fischer K (2007). Number sense and developmental dyscalculia. Human Behavior, Learning, and the Developing Brain: Atypical Development.

[B6] Dehaene S, Piazza M, Pinel P, Cohen L (2003). Three parietal circuits for number processing. Cogn Neuropsychol.

[B7] Molko N, Cachia A, Rivière D, Mangin J-F, Bruandet M, Le Bihan D, Cohen L, Dehaene S (2003). Functional and structural alterations of the intraparietal sulcus in a developmental dyscalculia of genetic origin. Neuron.

[B8] Isaacs EB, Edmonds CJ, Lucas A, Gadian DG (2001). Calculation difficulties in children of very low birthweight. A neural correlate. Brain.

[B9] Cohen Kadosh R, Cohen Kadosh K, Schumann T, Kaas A, Goebel R, Henik A, Sack AT (2007). Virtual dyscalculia induced by parietal-lobe TMS impairs automatic magnitude processing. Curr Biol.

[B10] Cantlon JF, Brannon EM, Carter EJ, Pelphrey KA (2006). Functional imaging of numerical processing in adults and 4-y-old children. PLoS Biol.

[B11] Rivera SM, Reiss AL, Eckert MA, Menon V (2005). Developmental changes in mental arithmetic: Evidence for increased functional specialization in the left inferior parietal cortex. Cereb Cortex.

[B12] Kaufmann L, Koppelstaetter F, Siedentopf C, Haala I, Haberlandt E, Zimmerhackl L-B, Felber S, Ischebeck A (2006). Neural correlates of a number-size interference task in children. NeuroReport.

[B13] Kaufmann L, Vogel S, Wood G, Kremser C, Schocke M, Zimmerhackl L-B, Koten JW (2008). A developmental fMRI study of nonsymbolic numerical and spatial processing. Cortex.

[B14] Kucian K, Loenneker T, Dietrich T, Dosch M, Martin E, von Aster M (2006). Impaired neural networks for approximate calculation in dyscalculic children: A functional MRI study. Behav Brain Funct.

[B15] Mussolin C, De Volder A, Grandin C, Schlögel X, Nassogne M-C, Noël M-P (2009). Neural correlates of symbolic number comparison in developmental dyscalculia. J Cogn Neurosci.

[B16] Kaufmann L, Vogel SE, Starke M, Kremser C, Schocke M (2009). Numerical and non-numerical ordinality processing in children with and without developmental dyscalculia: Evidence from fMRI. Cogn Dev.

[B17] Price GR, Holloway I, Räsänen P, Vesterinen M, Ansari D (2007). Impaired parietal magnitude processing in developmental dyscalculia. Curr Biol.

[B18] Tewes U, Rossmann P, Schallberger U (1999). Hamburg-Wechsler-Intelligenztest für Kinder III.

[B19] Haffner J, Baro K, Parzer P, Resch F (2005). Heidelberger Rechentest (HRT 1–4) Erfassung mathematischer Basiskompetenzen im Grundschulalter.

[B20] Walsh V (2003). A theory of magnitude: common cortical metrics of time, space and quantity. Trends Cogn Sci.

[B21] Talairach J, Tournoux P (1988). Co-planar stereotaxic atlas of the human brain.

[B22] Cohen Kadosh R, Lammertyn J, Izard V (2008). Are numbers special? An overview of chronometric, neuroimaging, developmental and comparative studies of magnitude representation. Progr Neurobiol.

[B23] Grabner RH, Ansari D, Reishofer G, Stern E, Ebner F, Neuper C (2007). Individual differences in mathematical competence predict parietal brain activation during mental arithmetic. NeuroImage.

[B24] Geary DC, Hoard MK, Byrd-Craven J, DeSoto MC (2004). Strategy choices in simple and complex addition: Contributions of working memory and counting knowledge for children with mathematical disability. J Exp Child Psychol.

[B25] Ansari D (2007). Does the parietal cortex distinguish between "10", "ten," and ten dots?. Neuron.

[B26] Cabeza R (2008). Role of parietal regions in episodic memory retrieval: The dual attentional processes hypothesis. Neuropsychologia.

[B27] Logothetis NK (2003). The underpinnings of the BOLD functional magnetic resonance imaging signal. J Neurosci.

[B28] Kaufmann L, Ischebeck A, Koppelstaetter F, Siedentopf C, Weiss E, Vogel SE, Gotwald T, Marksteiner J, Wood G (2008). An fMRI Study of the numerical Stroop task in individuals with and without minimal cognitive impairment. Cortex.

[B29] Yetkin FZ, Rosenberg RN, Weiner MF, Purdy PD, Cullum CM (2006). FMRI of working memory in patients with mild cognitive impairment and probable Alzheimer's disease. Eur Radiol.

